# Modified chair method: an easy and efficient reduction method without medication for anterior shoulder dislocation

**DOI:** 10.1186/s12873-022-00757-8

**Published:** 2022-12-05

**Authors:** Yufeng Ge, Minghui Yang, Feng Gao, Weidong Peng, Xinbao Wu

**Affiliations:** Department of Orthopaedics and Traumatology, Peking University Fourth School of Clinical Medicine, Beijing Jishuitan Hospital, Beijing, China

**Keywords:** Anterior shoulder dislocation, Closed reduction, Chair method

## Abstract

**Background:**

Various maneuvers have been introduced to address anterior shoulder dislocations. Chair method allows the patient to sit comfortably and feel less pain during the reduction procedure. However, the rarity of comparative studies led to a lack of evidence to popularize. The present study aimed to introduce a modified chair (MOC) reduction method for anterior shoulder dislocation and explore its effectiveness compared with the traditional Hippocratic approach.

**Methods:**

This is a single-center retrospective study of 257 patients with anterior shoulder dislocation from September 2020 and July 2021. Patients were divided into two groups according to the reduction method they received (either the Hippocratic method or the MOC method). Success rate, reduction time, visual analog scale (VAS) pain score, satisfaction level, and a new indicator, pain index (reduction time (s)* VAS/ 10), were compared.

**Results:**

One hundred sixteen patients (43 females, 73 males) underwent the Hippocratic method, and 141 (65 females, 76 males) MOC method. A significantly higher success rate was seen in the MOC group (96.5%(136/141) vs. 84.5%(98/116) in the Hippocratic group; OR 5, 95%CI 1.79 ~ 13.91; *p* = 0.002). Pain index of the patients in the MOC group was much lower than that in the Hippocratic group (3.20 (2.10, 4.53) vs. 36.70 (22.40, 47.25), *p* <  0.001). The reduction time, VAS pain score, and satisfaction level also favored the MOC method.

**Conclusions:**

The MOC method is an easy and efficient reduction method with minimum assistance for anterior shoulder dislocations. Physicians can skillfully perform this procedure with the help of their body weight. The MOC method could be attempted for shoulder dislocations in the emergency department.

## Background

Acute shoulder dislocation is a widespread problem encountered in the Emergency Department (ED) [1]. There are several methods of reduction, some of which require the patient to lie supine, for example, the Kocher maneuver [2], the Hippocratic way [3], and FARES (Fast, Reliable, and Safe) [4]. Some require the prone position, such as the Stimson technique [5] and the scapular manipulation [6]. Both supine and prone positions demand the patients, who walk into the emergency room in most cases, to lie down. This posture-changing could be very painful and time-consuming, let alone the Stimson method requiring 20mins to achieve the reduction [5].

White [7] first introduced a chair method in 1976, and then Westin [8] described his snowbird technique that required little analgesia. In this century, a specialized reduction chair (Oxford chair) for shoulder dislocation was designed [9]. Patients undergoing this sitting procedure felt relatively less pain, and the total reduction time was much shorter when compared with other methods [9, 10]. However, the force applied in these reduction maneuvers still originates from the muscle strength of the physicians, who often get exhausted after a few minutes. Besides, the reported success rates ranged from 62 to 100% [8–13]. Comparative studies involving the chair method were rare, resulting in a lack of evidence to popularize this efficient method.

The present study aimed to introduce a modified chair (MOC) reduction method for anterior shoulder dislocation and explore its effectiveness compared with the traditional Hippocratic way.

## Methods

### Design and participant selection

Between September 2020 and July 2021, this retrospective study enrolled 302 patients aged 14 years or over and presented with anterior shoulder dislocation in the emergency department in a level-1 trauma center. Patients who were treated with either the Hippocratic method or MOC method were included. The exclusion criteria included multiple trauma, noncooperation (as it may complicate the procedure), and concomitant glenoid fracture or surgical neck fracture of the proximal humerus. Patients who asked for anesthesia or oral analgesics prior to the reduction maneuver were also excluded. All patients gave written informed consent before reduction, and the Institutional Review Board approved the study of the Beijing Jishuitan Hospital (Approval no.: 202112–06).

Anteroposterior (AP) and lateral radiographs were obtained from all patients before and after the reduction to confirm the diagnosis, and neurovascular status was reassessed. Computed Tomography (CT) was also obtained if necessary to exclude occult surgical neck fracture. Reduction procedures were performed by six junior residents who were in their second or third year in orthopedic surgery. Three junior residents were trained for MOC methods, while three were for the Hippocratic method. Patients were divided into two groups according to the reduction method (Hippocratic or MOC group).

### Reduction technique

**Fig. 1:**
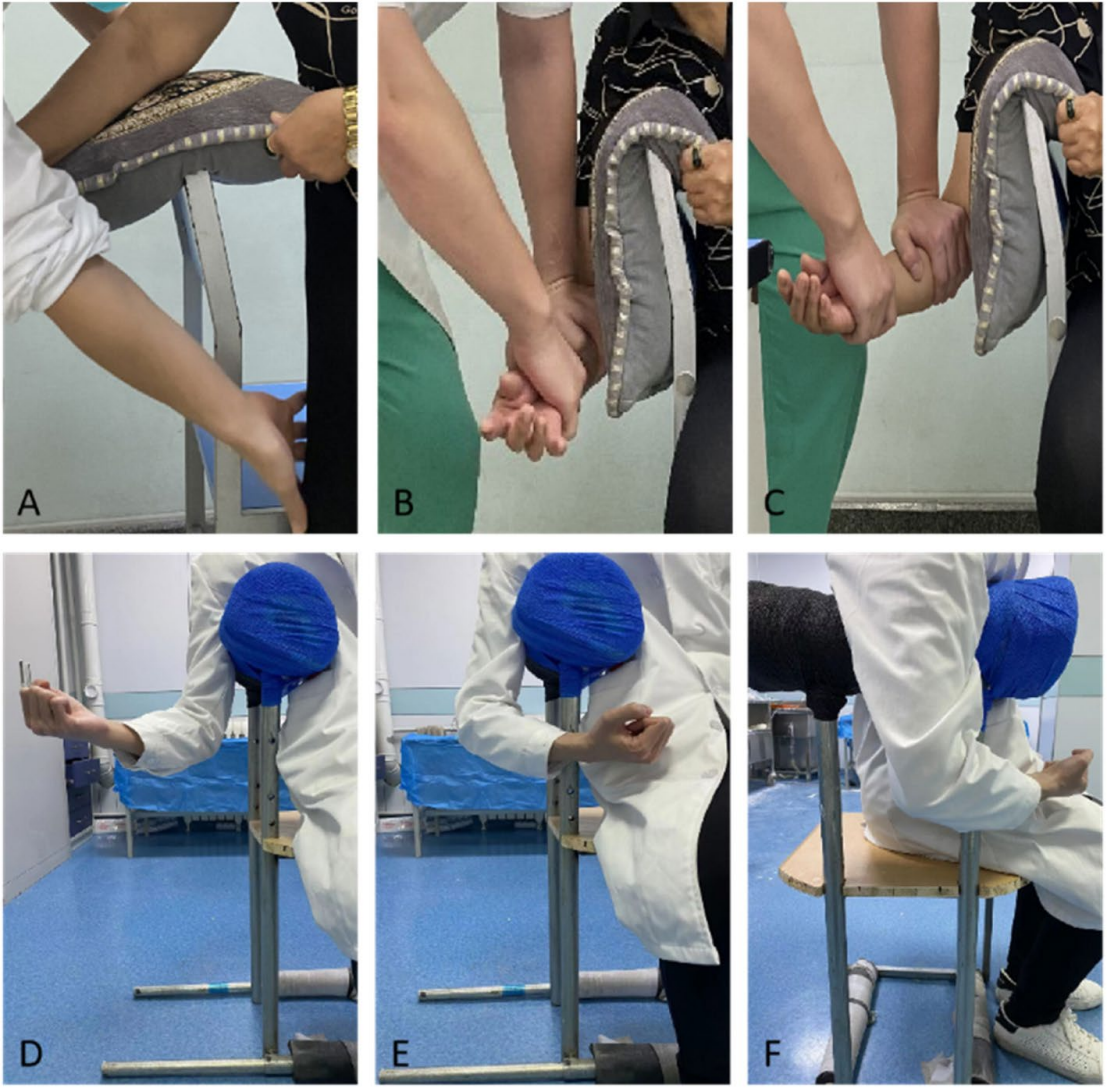
Modified Chair (MOC) method of reduction for shoulder dislocation. **A** Patients slightly abducted the upper arm and used the backrest of the chair as a fulcrum in the axilla; **B** Downward traction without external rotation was applied by the physician with his left elbow extended; **C** External rotation was added during traction; **D-F** Illustration of external and internal rotation on a specialized chair made for MOC method

MOC method is performed with the patient sitting in a chair sideways. The patient is guided to abduct the upper arm slightly and then use the chair’s backrest as a fulcrum in the axilla. In order to maximize the comfort level and minimize the risks of nerve injury, a pillow or cushion or folded clothing is used as a pad on the backrest (Fig. 1A). With the padding, the height of the chair’s back could be adjusted individually. The dislocated arm is allowed to hang over the backrest and passively flexed at the elbow. The physician holds the patient’s forearm or wrist with the right hand (for right shoulder dislocations) and places the left palm at the proximal forearm (Fig. 1B). Downward traction is then applied slowly with the physician’s left elbow extended. During this procedure, the bodyweight of the physician, not the strength of muscles in other methods, is used as the main force, and different degrees of external rotation (ER) could be easily applied (Fig. 1C). Once reduction is achieved after feeling and/or hearing a clunk, the physician could conveniently inspect and palpate the affected shoulder and then internal-rotate it while the patient is still sitting. A simple chair was then made to facilitate such a maneuver (Fig. 1D-F).

The Hippocratic method was conducted as in other literature described before [4, 14]. Countertraction was exerted either by assistants utilizing a sheet or by the physician’s heel placed at the axilla of the patients.

In patients who failed two attempts with the same reduction maneuver, we recorded it as failures and employed other reduction methods or further anesthesia like sedation and nerve block to accomplish the reduction procedure. All patients were immobilized in internal rotation with a shoulder/arm sling for 4 weeks. After 4 weeks, the patients underwent rehabilitation.

### Data collection and Outcome measures

Data regarding demographic information, injury mechanism, the time interval between injury and presentation, history of the previous dislocation, neurovascular assessment, the presence of a greater tubercle fracture, reduction method, reduction time, success or not, complications, visual analog scale (VAS) and satisfaction level were recorded and collected for all patients. In our study, we introduced a variate called pain index, taking both reduction time and pain score into account to judge the efficiency of a reduction maneuver (pain index = reduction time (s)* VAS/ 10), and the higher the pain index, the worse the outcome).

The success rate and pain index were regarded as the primary outcome. The duration of reduction was accepted as the time between the patient being placed in the reduction position and the completion of reduction. The time was recorded with a stopwatch. VAS from 0 (no pain) to 10 (intolerable pain) was used to rate the amount of pain felt during the process. Satisfaction level was documented as very dissatisfied, dissatisfied, general, satisfied, and very satisfied. The same orthopedic surgeon who had performed the reduction asked and assessed the patients VAS pain scores and satisfaction level.

### Data analysis

Missing data in the time interval between injury and reduction (33(13%) were randomly missing, 8(7%) and 25(18%) in the Hippocratic group and MOC group, respectively) were filled with the median. Data are presented as means and standard deviations for parametric data or as medians and interquartile ranges when the data are not normally distributed. Categorical variables are described using frequencies and numerical distributions. The Chi-squared test was used to assess the differences between the two groups for categorical variables and Student’s t-test or the Mann-Whitney U-test for continuous variables, as appropriate (parametric vs. non-parametric data, respectively). With pain index as the outcome, stratified analysis was further conducted according to age grouping (above and below 60 years), sex, the time interval between injury and reduction (over and within 6 hours), recurrent dislocation, and fracture of the greater tubercle. The analyses were performed with the statistical software packages R (http://www.R-project.org, The R Foundation) and Free Statistics software version 1.4. A two-tailed test was performed, and *p* <  0.05 was considered statistically significant.

## Results

**Fig. 2:**
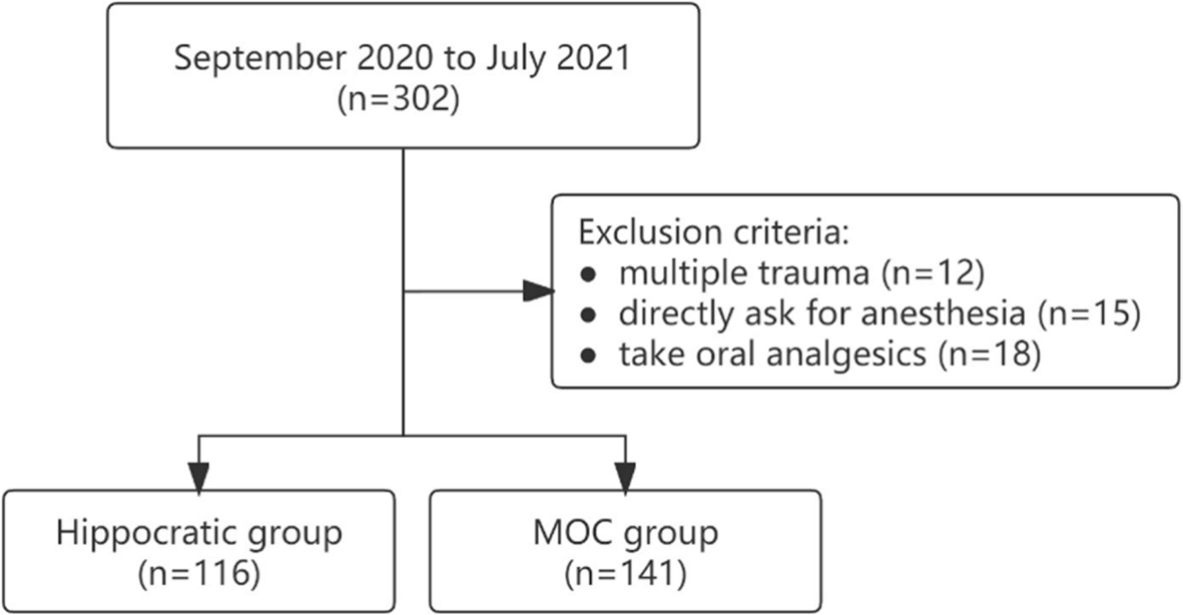
Study flowchart

In our study, a total of 257 patients (108 females, 149 males; mean age, 52.13 years; age range, 15–90 years) were ultimately compared. The flow is shown in Fig. 2. 116 patients (43 females, 73 males) were treated with the Hippocratic method, and 141 (65 females, 76 males) with the MOC method. The baseline characteristics were similar and comparable between these two groups (Table 1). The baseline characteristics were shown in Table 1. Ninety-one (35.4%) of patients sustained a concomitant fracture of the greater tubercle. Three patients with distal radius fractures that could not undergo the Hippocratic method were reduced through the MOC approach, and all three were successful with slight pain. No complications or neurovascular injuries were noted following the reduction.

**Table 1:** Baseline characteristics of patients

	Total (***n*** = 257)	Hippocratic Method (***n*** = 116)	MOC Method (***n*** = 141)	***p*** Value
Age (years, mean, SD)	52.13 ± 20.10	50.06 ± 20.79	53.83 ± 19.42	0.135
Sex (n,%)				0.183
Female	108 (42.0)	43 (37.1)	65 (46.1)	
Male	149 (58.0)	73 (62.9)	76 (53.9)	
Recurrent dislocation (n,%)				0.38
No	213 (82.9)	93 (80.2)	120 (85.1)	
Yes	44 (17.1)	23 (19.8)	21 (14.9)	
Mechanism of injury (n,%)				0.473
Sports activity	31 (12.1)	14 (12.1)	17 (12.1)	
Fall	189 (73.5)	87 (75)	102 (72.3)	
Traffic accident	7 (2.7)	1 (0.9)	6 (4.3)	
Non-violence^a^	30 (11.7)	14 (12.1)	16 (11.3)	
Time interval between injury and reduction (hours, Median, IQR)	3.00 (2.00, 6.00)	3.00 (2.00, 6.00)	3.00 (1.00, 6.00)	0.526
Fracture of greater tubercle (n,%)				0.88
No	166 (64.6)	76 (65.5)	90 (63.8)	
Yes	91 (35.4)	40 (34.5)	51 (36.2)	

^a^Non-violence included behavior like lifting a heavy object, shaking hands, seizures, or turning over during sleep

**Fig. 3:**
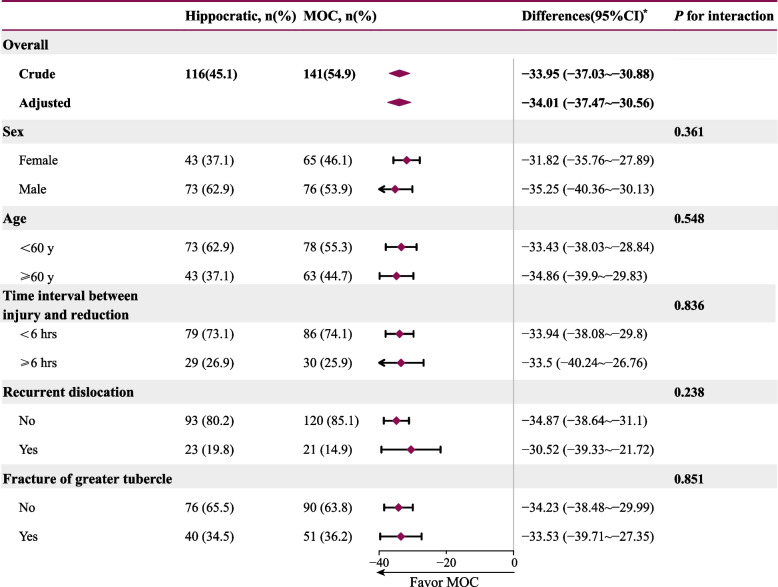
Stratified linear analysis of pain index difference between two methods (pain index = reduction time (s)* VAS/ 10). ^*^ Hippocratic group’s pain index as the reference, and the results were adjusted for age, sex, and the time interval between injury and reduction

A significantly higher success rate was seen in the MOC group (96.5%(136/141) vs. 84.5%(98/116) in the Hippocratic group; OR 5, 95%CI 1.79 ~ 13.91; *p* = 0.002) (Table 2). The pain index of the patients in the MOC group was much lower than that in the Hippocratic group (3.20 (2.10, 4.53) vs. 36.70 (22.40, 47.25), *p* <  0.001) (Table 2). The reduction time, VAS pain score, and satisfaction level were also in favor of the MOC method, and the differences were significant (*p* <  0.001) (Table 2). The minimum reduction time spent in the MOC group was only 3 seconds. In addition, the stratified linear analysis results also supported the effectiveness of the MOC method in different patient populations (Fig. 3).

**Table 2:** Outcome parameters compared between two methods

	Total (***n*** = 257)	Hippocratic Method (***n*** = 116)	MOC Method (***n*** = 141)	***p*** Value
Success rate (n,%)	234 (91.1)	98 (84.5)	136 (96.5)	0.002
Pain Index^a^ (Median, IQR)	5.15 (2.80, 29.75)	36.70 (22.40, 47.25)	3.20 (2.10, 4.53)	< 0.001
Duration of reduction (seconds, Median, IQR)	20.00 (11.00, 64.00)	71.00 (52.25, 94.75)	12.00 (9.00, 15.25)	< 0.001
VAS pain score (Median, IQR)	3.00 (3.00, 5.00)	6.00 (4.00, 6.00)	3.00 (2.00, 3.00)	< 0.001
Satisfactory level (n,%)				< 0.001
Very dissatisfied	30 (11.7)	30 (25.9)	0 (0)	
Dissatisfied	24 (9.3)	24 (20.7)	0 (0)	
General	36 (14.0)	20 (17.2)	16 (11.3)	
Satisfied	88 (34.2)	28 (24.1)	60 (42.6)	
Very satisfied	79 (30.7)	14 (12.1)	65 (46.1)	

*Abbreviations*: *VAS* Visual analog scale

^a^Pain Index = VAS pain score

^b^Duration of reduction(s)/10

## Discussion

In this present retrospective study, a comparison between the MOC method and the Hippocratic method was conducted to assess its effectiveness and safety. The study results demonstrated that the MOC method had an advantage over Hippocratic in terms of all the outcome parameters, especially in the pain index, which combined the two biggest concerns of dislocated patients - time and the VAS pain score. In the MOC group, 105/141(74.5%) patients felt a pain level of less than 4, indicating the reason behind the phenomenon that 125/141(88.7%) patients felt more than satisfied.

The reduction time in the MOC group ranged from 3 s to 30s with a median of 12 s, which was significantly shorter than the Hippocratic group (median 73 s, range from 16 s to 119 s, *p* < 0.001). Ten patients (7.1%) received reduction within 5 s. This is the shortest duration of reduction ever reported to the best of our knowledge. Mahiroğulları [12] reported their mean duration of reduction as 13.9 (range: 3 to 45) seconds in 74 patients undergoing the chair method and a success rate of 100%, like our results (success rate 96.5% (136/141)). Guler et al. [10] retrospectively compared four different methods in 153 patients, demonstrating a shorter reduction time in the chair method group than in other methods. However, in their studies, we did not find a detailed description of time-keeping. In our study, we started clocking once patients got ready. Physicians using the MOC method could directly place their hands on patients’ already prepared forearms, while the Hippocratic method required physicians to add counter traction either by sheet wrapping around the patients or by well-positioning their heels at the patients’ axilla. Sometimes bandages were needed to wrap around the patients’ wrists or hands to increase the friction force. The time cost for such posture or extra maneuver during the reduction in the Hippocratic group was also counted in the duration of reduction, which explained part of the significant differences. Guler [10] reported the success rate in the chair group of 97.8% (46/47), as in our results.

MOC method allowed the patients, who walked into the emergency room in most cases, to place themselves in a sitting posture. Instead of lying down described in many other methods, the sitting position spared the patients from changing posture, which is painful and time-consuming [8, 9]. In our experience, patients undergoing the MOC method made quicker preparation before reduction, but such time was not collected in this present study. Chung [9] reported a 21 min shorter length of stay in ED for the chair group (Oxford chair) in comparison with the traditional method group (Kocher’s maneuver). Moreover, patients were relatively pain-free in such a sitting position where the affected limb could lie over the back of the chair with the pillow in the axilla and the arms hanging. This could partly explain the significantly lower VAS score and higher satisfaction level rate in the MOC group. In Mahiroğulları [12] ‘s study, all the patients undergoing the chair method answered the question, “Would you like your shoulder to be relocated using this method if it dislocates again? “as “Yes.” Those answers and the differences in satisfaction rate in our study revealed the potential comfort in the chair method.

Three reduction techniques (traction-counter traction, leverage, scapular manipulation) were concluded and compared in a meta-analysis conducted by Dong et al. [15]. However, the chair method was not included and categorized in these three techniques. In the MOC method, with the physician’s elbow extended, gradually increased downward force was applied to the elbow while evaluating the pain level patient felt by asking. The traction force applied straight originated from the physician’s body weight in line with the gravity, not the strength of the physician’s muscles. The posture resembles that in CPR (cardiopulmonary resuscitation, CPR). Moreover, unlike the force conduction in Hippocratic or other chair methods relying on friction, the traction was directly transmitted to the shoulder, maximizing the use of body weight and making reduction easier. We thought this was the main reason for the shorter time in the MOC method. In this way, we believe the MOC method could help physicians improve their efficiency in busy emergency work and possibly avoid physical exhaustion, especially in the case of a muscular patient who may be considered for anesthesia. Sometimes external rotation (ER) (leverage techniques) was needed to dislodge the trap of the humeral head [16]. The patient’s posture in MOC resembled the position during checking rotation of the shoulder, so another advantage of the MOC method was that it allowed the physicians to apply a certain degree of ER and document it, facilitating the reduction procedure (Fig. 1C). In methods requiring an extended elbow like Hippocratic or Spaso, most of the rotational force applied distally only results in supination and pronation of the forearm. Last, the scapula was relatively fixed by asking the patients to lean against the chair back as tight as possible. Therefore, combined techniques were utilized in the MOC method, and traction and ER were much easier to apply in such a way without other assistance. We then developed a simple chair personalized for shoulder dislocations in our Emergency Department (Fig. 1D-F).

Muscle contraction was often reported to cause difficulty, pain, and even iatrogenic fracture during reduction [17, 18]. In our study, the chair’s backrest helped prevent the patients from contracting their muscles, and none of the patients had any such complications. Here are two tips we proved helpful in relaxing muscles during the MOC method. First, slowly increase the traction force while asking about patients’ pain level during reduction. This could assist patients in relaxing and adapting for a while. Second, ask the patient to clench the fist of the affected limb and then redo it while counting the number of times. We found that the muscle could relax when patients performed such action and focused on it. Anesthesia or sedation was almost spared due to MOC’s simplicity, rapidity and relative free of pain.

The main limitation of the present study was its retrospective design. Lack of quality control, like data missing, was inevitable, resulting in selection bias. However, the missing data in our study were randomly distributed, and the sample size was large enough to reflect the differences. Another limitation is that some of the patients came to our hospital after several failed attempts in other hospitals. This kind of data was not documented and collected due to its retrospective nature as well. In addition, the pain index introduced in our study was an attempt to reflect both aspects of reduction time and pain level, and to some degree, it may overstate the differences between the two methods. Future studies could further explore the value of such an indicator. Last, we only compared the MOC method with the Hippocratic method because these two were the main reduction methods conducted in our ED. Further well-designed prospective trials are needed to compare MOC with other methods. Further well-designed prospective trials are required to compare MOC with other methods to better evaluate its effectiveness.

## Conclusions

In conclusion, the MOC method is an easy and efficient reduction method with minimum assistance for anterior shoulder dislocations. Physicians can skillfully perform this procedure with the help of their body weight. The MOC method could be attempted for shoulder dislocations in the emergency department.

## Data Availability

The datasets used in this study are not publicly available because of patient confidentiality but are available from the corresponding author on reasonable request.

## Abbreviations

ED: Emergency department
MOC: Modified chair
AP: Anteroposterior
CT: Computed tomography
ER: External rotation
VAS: Visual analog scale
CPR: Cardiopulmonary resuscitation

## References

[1] Owens BD, Duffey ML, Nelson BJ, DeBerardino TM, Taylor DC, Mountcastle SB (2007). The incidence and characteristics of shoulder instability at the United States Military Academy. Am J Sports Med.

[2] Uglow MG (1998). Kocher’s painless reduction of anterior dislocation of the shoulder: a prospective randomised trial. Injury.

[3] Mattick A, Wyatt JP (2000). From Hippocrates to the Eskimo--a history of techniques used to reduce anterior dislocation of the shoulder. J R Coll Surg Edinb.

[4] Sayegh FE, Kenanidis EI, Papavasiliou KA, Potoupnis ME, Kirkos JM, Kapetanos GA (2009). Reduction of acute anterior dislocations: a prospective randomized study comparing a new technique with the Hippocratic and Kocher methods. J Bone Joint Surg Am.

[5] Stimson LA (1900). An easy method of reducing dislocations of the shoulder and hip. Med Rec.

[6] Sahin N, Ozturk A, Ozkan Y, Atici T, Ozkaya G (2011). A comparison of the scapular manipulation and Kocher’s technique for acute anterior dislocation of the shoulder. Eklem Hastalik Cerrahisi.

[7] White AD (1976). Dislocated shoulder-a simple method of reduction. Med J Aust.

[8] Westin CD, Gill EA, Noyes ME, Hubbard M, Anterior shoulder dislocation. (1995). A simple and rapid method for reduction. Am J Sports Med.

[9] Chung JY, Cheng CH, Graham CA, Rainer TH (2013). The effectiveness of a specially designed shoulder chair for closed reduction of acute shoulder dislocation in the emergency department: a randomised control trial. Emerg Med J.

[10] Guler O, Ekinci S, Akyildiz F, Tirmik U, Cakmak S, Ugras A (2015). Comparison of four different reduction methods for anterior dislocation of the shoulder. J Orthop Surg Res.

[11] Noordeen MH, Bacarese-Hamilton IH, Belham GJ, Kirwan EO (1992). Anterior dislocation of the shoulder: a simple method of reduction. Injury.

[12] Mahirogullari M, Akyildiz F, Koksal I, Cakmak S, Kurklu M, Kuskucu M (2012). Chair method: a simple and effective method for reduction of anterior shoulder dislocation. Acta Orthop Traumatol Turc.

[13] Smith SL (2009). An investigation comparing the Oxford Chair Technique with the traditional methods of glenohumeral dislocation reduction currently implemented. Int Emerg Nurs.

[14] Alkaduhimi H, van der Linde JA, Flipsen M, van Deurzen DF, van den Bekerom MP (2016). A systematic and technical guide on how to reduce a shoulder dislocation. Turk J Emerg Med.

[15] Dong H, Jenner EA, Theivendran K (2021). Closed reduction techniques for acute anterior shoulder dislocation: a systematic review and meta-analysis. Eur J Trauma Emerg Surg.

[16] Cunningham NJ (2005). Techniques for reduction of anteroinferior shoulder dislocation. Emerg Med Australas.

[17] Pan X, Yao Y, Yan H, Wang J, Dai L, Qu X (2021). Iatrogenic fracture during shoulder dislocation reduction: characteristics, management and outcomes. Eur J Med Res.

[18] Leroux T, Wasserstein D, Veillette C, Khoshbin A, Henry P, Chahal J (2014). Epidemiology of primary anterior shoulder dislocation requiring closed reduction in Ontario, Canada. Am J Sports Med.

